# Tactile and olfactory stimulation reduce anxiety and enhance autonomic balance: a multisensory approach for healthcare settings

**DOI:** 10.1186/s40359-025-03140-x

**Published:** 2025-07-18

**Authors:** Junfang Xie, Mohamed Elsadek, Zhang Deshun, Zhiyi Zhou, Jie Gao

**Affiliations:** 1 Department of Urban and Rural Planning, College of Architecture and Design, Nanchang University, Jiangxi, 330031 China; 2 Department of Landscape Architecture, College of Architecture and Urban Planning, Tongji University, Shanghai, 200092 China; 3 Department of Horticulture, Faculty of Agriculture, Suez Canal University, Ismailia, 41522 Egypt; 4 Shanghai Xiaofeng Dental Clinic Group, Shanghai, China

**Keywords:** Multisensory stimulation, Biophilic design, Dental anxiety, Heart rate variability, Skin conductance, Parasympathetic activation, Anxiety reduction, Dental clinic environment

## Abstract

Anxiety in healthcare environments—particularly in dental clinics—presents a significant challenge, often impairing patient cooperation and clinical outcomes. This study investigated the effectiveness of multisensory engagement—combining visual, tactile, and olfactory stimuli—in reducing anxiety and promoting physiological relaxation within a dental clinic setting. A within-subject experimental design was employed, exposing 40 participants to five conditions: control (no sensory input), visual (plant observation), tactile (plant interaction), olfactory (plant scent), and combined tactile-olfactory stimulation. Anxiety was assessed using the State-Trait Anxiety Inventory– State (STAI–S), and physiological responses were measured via heart rate (HR), heart rate variability (HRV) indices (LF/HF ratio, RMSSD, SDNN), and skin conductance (SC). Findings revealed significant reductions in anxiety and stress markers across all sensory conditions compared to the control. The tactile-olfactory combination elicited the most consistent improvements across physiological indices, including a 42.3% reduction in STAI–S scores, a 66.0% decrease in the LF/HF ratio, 81.6% increase in RMSSD (olfactory condition peak), a 54.7% increase in SDNN, a 15.9% decrease in HR, and a 67.2% reduction in SC. These findings underscore the potential of nature-based multisensory interventions to enhance autonomic regulation and psychological well-being in clinical environments. The study offers novel insights into the application of biophilic design principles in healthcare, demonstrating that engaging multiple sensory pathways, especially combined tactile and olfactory inputs, can help mitigate patient anxiety and promote relaxation.

## Introduction

Anxiety and stress-related disorders are among the most prevalent mental health challenges globally, significantly affecting patient outcomes and healthcare systems [1]. In clinical settings such as hospitals and outpatient clinics, heightened anxiety is linked to poorer patient cooperation, delayed recovery, and increased healthcare costs [2]. Beyond the psychological impact, anxiety triggers physiological responses, such as overactivation of the sympathetic nervous system, impairing healing and reducing treatment efficacy [3]. Therefore, addressing anxiety is critical not only for improving patient well-being but also for enhancing compliance and optimizing clinical outcomes.

Among healthcare-related stressors, dental anxiety is particularly widespread and debilitating, affecting a significant portion of the global population. Characterized by intense fear of dental procedures and the clinical environment, dental anxiety often leads to avoidance of treatment, resulting in deteriorating oral health and a cyclical pattern of increased fear and worsened health outcomes [4–6]. This heightened anxiety is often triggered by sensory stimuli, such as the sound of drills, the sight of instruments, and the perceived invasiveness of dental procedures, making dental clinics uniquely stressful environments [7]. Consequently, innovative, evidence-based strategies are urgently needed to alleviate anxiety and improve patient comfort in these settings.

Physiological markers, such as heart rate variability (HRV) and skin conductance (SC) provide objective measures of stress and relaxation. HRV, which reflects the balance between the parasympathetic and sympathetic branches of the autonomic nervous system, is widely recognized as a reliable indicator of stress adaptability. Higher HRV indicates relaxation and adaptability, while lower HRV reflects heightened stress and sympathetic dominance [8]. Similarly, SC measures changes in sweat gland activity, offering real-time insights into sympathetic arousal [9]. When combined with psychological assessments, such as the State-Trait Anxiety Inventory (STAI–S), these markers offer a comprehensive framework for evaluating sensory interventions in clinical settings.

Emerging research highlights the potential of multisensory engagement to reduce anxiety by integrating sensory inputs—such as vision, smell, and touch—to promote psychological well-being and autonomic regulation [10]. Human perception relies on the integration of sensory inputs to form a cohesive understanding of the environment. While sensory modalities are broadly categorized into distance senses (e.g., vision, hearing, and smell) and proximity senses (e.g., touch and taste), each plays a distinct role in shaping emotional responses [11, 12]. Fechner’s theory of aesthetic appreciation emphasizes the significance of multisensory engagement, positing that combining multiple sensory stimuli can enhance emotional responses and well-being [13]. Despite this understanding, healthcare settings often remain sterile and visually monotonous, inadvertently exacerbating stress and anxiety among patients.

Plants provide several proven benefits in indoor settings, such as air purification [14], thermal regulation, and psychological support. Incorporating natural elements into clinical environments can further engage multiple senses, promoting relaxation and enhancing overall well-being [15, 16]. For example, visual exposure to greenery is associated with reduced heart rates and cortisol levels, while tactile and olfactory interactions with natural elements amplify these calming effects [17–22]. Recent findings suggest that combining sensory modalities, such as visual, tactile, and olfactory stimuli, enhances stress reduction and well-being compared to single-modality interventions. For instance, Qu and Ma demonstrated that multisensory engagement in urban green spaces significantly enhances psychological well-being and stress recovery [23]. However, much of this research has been conducted in outdoor or virtual environments [24, 25],, leaving its application to indoor healthcare settings, particularly dental clinics, underexplored.

Building on these findings, nature’s benefits extend beyond the visual sense to include profound yet underexplored contributions from tactile and olfactory modalities. Specifically, the effects of tactile and smell interaction with natural elements, such as plants, are notably underrepresented in the literature, underscoring the urgent need for further investigations to uncover their potential in enhancing psychological well-being, particularly in indoor environments [26].

Multisensory engagement, which integrates visual, tactile, olfactory, and auditory stimuli, has emerged as a promising approach to creating immersive environments that promote relaxation and well-being [22]. Supporting this, Song et al. demonstrated that virtual urban green environments combining these multisensory stimuli significantly enhance physiological and psychological recovery. While prior studies have predominantly focused on single-modality interventions, such as visual or auditory stimuli, there is a growing recognition of the restorative potential of tactile and olfactory inputs in promoting relaxation and emotional balance [27, 28].

Theoretical frameworks, such as Stress Reduction Theory (SRT) and Attention Restoration Theory (ART), provide valuable insights into the mechanisms underlying these effects. SRT posits that exposure to natural elements promotes physiological relaxation, by activating parasympathetic activity, while ART emphasizes the restorative power of nature in alleviating mental fatigue and restoring cognitive capacities [29, 30]. Although visual or auditory inputs have traditionally been emphasized [17, 31–33], recent evidence highlights the critical role of other sensory modalities, in maximizing the restorative benefits of sensory-enriched environments [34]. These frameworks suggest a strong potential for nature-inspired, multisensory interventions in healthcare settings, yet their application to anxiety reduction in dental clinics remains insufficiently studied.

This study addresses these gaps by investigating the combined effects of visual, tactile, and olfactory stimuli on anxiety and stress in a dental clinic setting. By measuring physiological markers such as HRV and SC, alongside psychological assessments using the STAI–S, this research aims to evaluate the effectiveness of multisensory interventions in promoting relaxation and reducing anxiety. Specifically, this study has three objectives: (1) to assess the individual and combined effects of visual, tactile, and olfactory stimuli on anxiety and stress reduction; (2) to provide actionable insights for integrating multisensory, nature-based interventions into clinical environments; and (3) to contribute to the growing evidence supporting biophilic design and sensory engagement in healthcare settings.

We hypothesize that (i) Participants exposed to multisensory engagement (e.g., tactile–olfactory plant stimuli) will report significantly greater environmental comfort and satisfaction compared to those exposed to single-modality sensory conditions or control, (ii) these effects will be mediated by enhanced parasympathetic activation. The findings are expected to inform best practices for designing supportive, patient-centered spaces in dental clinics and other high-stress healthcare environments. By demonstrating the potential of multisensory engagement, this study offers a non-invasive, scalable solution to a pervasive healthcare challenge.

## Materials and methods

### Participants and recruitment

Forty volunteers (20 males and 20 females) with a mean age of 24.32 ± 4.00 years participated in this study (see Table 1). All participants had normal or corrected-to-normal vision and met the inclusion criteria of no medication use, no intake of caffeinated or alcoholic beverages within 24 h prior to the experiment, and avoidance of strenuous physical activity within 2 h of the study. The adequacy of the sample size was determined using G*Power (version 3.1.9.7) for a two-tailed, paired t-test (within-subjects design). We assumed a medium effect size (Cohen’s d = 0.5), with α = 0.05, and statistical power (1–β) = 0.8. Based on these parameters, the minimum required sample size was calculated as 34 participants. Our final sample of 40 participants exceeds this requirement, ensuring sufficient power (> 80%) to detect medium within-subject effects across the sensory conditions.

**Table 1:** Participant characteristics

Gender	*N*.	Age Mean ± SD	Height (cm)	Weight (kg)	BMI (kg/m²)
Male	20	25.45 ± 4.41	171.95 ± 3.70	73.63 ± 3.56	24.93 ± 1.50
Female	20	23.21 ± 3.27	165.37 ± 3.42	56.47 ± 3.08	20.66 ± 1.21
Total	40	24.32 ± 4.00	168.66 ± 4.84	65.05 ± 9.29	22.79 ± 2.55

Participants were recruited using two approaches. First, study information was shared with clinic doctors, who then distributed the recruitment advertisement to their patients via WeChat groups, a widely used, semi-private social media platform in China. Second, the authors directly invited patients to participate during their clinic visits. Recruitment was based on participants’ availability and willingness to take part in the study. All experimental sessions were conducted in a fully operational dental clinic in Shanghai, using one of its consultation rooms to ensure high ecological validity.

Importantly, all participants were individuals who had scheduled appointments for standard dental treatment. They completed the experimental session while naturally waiting for their appointments. This timing allowed the study to capture anxiety levels in a realistic pre-treatment context. To minimize disruption, each trial was scheduled to align with the participant’s existing follow-up or check-up appointment. All clinical visits involved non-invasive procedures such as routine dental cleaning, assessments, or minor restorative treatments—no surgical interventions were involved during data collection.

All participants provided written informed consent prior to their involvement in the study. Additionally, informed consent was obtained for the use of any photographs or information that might potentially identify individuals. The study protocol conformed to the principles outlined in the Declaration of Helsinki and received ethical approval from the Ethics Committee of Tongji University (Approval No.: tjdxsr071).

### Experimental stimuli conditions

**Fig. 1:**
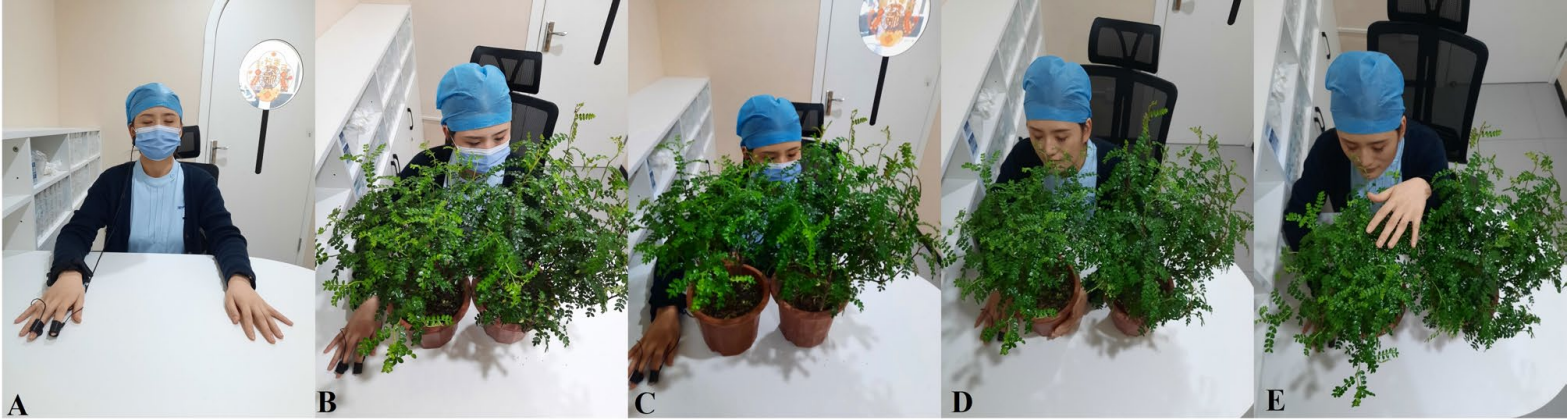
Experimental stimuli: **A**: control, **B**: Visual, **C**: Tactile, **D**: Olfactory, and **E**: Tactile-Olfactory stimuli. The figure includes a photograph of one of the assistants, provided to illustrate the study conditions

Green money plants (*Zanthoxylum piperitum*), native to Sichuan province and widely distributed across East Asia, were selected for their availability, aesthetic appeal, and distinct sweet, highly aromatic foliage. These features make them a suitable candidate for investigating the potential effects of olfactory interventions in a clinical setting. To ensure consistent exposure, two potted plants were positioned directly in front of each participant during the experiment, as illustrated in Fig. 1. This setup enabled visual, olfactory, and tactile interaction, ensuring a uniform multisensory experience across conditions.

### Experimental design and procedure

The experiment was conducted daily between 9:00–11:30 AM and 1:00–4:30 PM in a climate-controlled dental clinic room located in Shanghai. Environmental conditions were standardized: room dimensions were 2 m × 3.5 m × 3 m, temperature was maintained at 22 °C, relative humidity at 52%, and illumination at 300 lx.

A within-subject design was employed, allowing each participant to experience all five experimental conditions:

- Control: Participants were seated quietly without any sensory stimuli. This condition simulated a standard dental clinic environment.
- Visual: Participants viewed the plants placed 60 cm in front of them while wearing a mask to block olfactory input.
- Tactile: Participants touched and gently explored the texture of the plants (leaves, stems) with hands while keeping their eyes closed and wearing a mask. They were instructed to focus on the texture and temperature of the plants.
- Olfactory: Participants were instructed to inhale naturally while being exposed to the aroma of the plants. The plants were placed directly in front of the participant, who was seated with eyes closed to eliminate visual input.
- Tactile-Olfactory: Participants both touched and smelled the plants with their eyes closed. Instructions were to explore the plant’s texture and inhale the natural scent without exaggerating their breathing pattern.

Each condition lasted 4 min, a duration aligned with prior validations demonstrating that key metrics—RMSSD, SDNN, and frequency-domain indices (LF, HF, LF/HF)—can reliably be estimated from recordings of 90–240 s under controlled conditions [35–37]. Prior to the experiment, participants attended a standardized briefing to familiarize themselves with the study objectives and procedures. Upon entering the room, they were fitted with ErgoLAB wireless sensors for continuous physiological monitoring. Baseline anxiety levels were assessed using the STAI–S, followed by a 2-minute rest period with eyes closed to stabilize mood.

Each participant received a unique, computer-generated random sequence, ensuring that the distribution of condition orders was not systematically biased across the sample. We verified that all conditions, especially the Control, were evenly represented in different order positions across participants. Following each stimulus condition, participants completed the STAI–S questionnaire, resulting in five distinct anxiety assessments throughout the session. A 2-minute rest period was included between conditions to mitigate fatigue and prepare participants for the subsequent stimulus exposure.

**Fig. 2:**
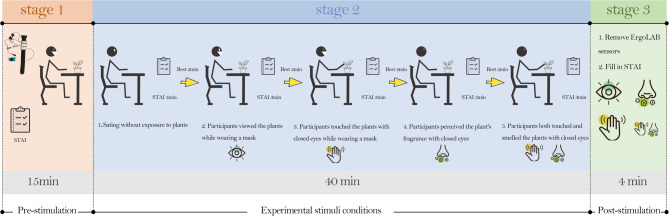
Study procedures

Figure 2. Experimental Procedure Overview. The protocol comprised three phases:

i. Pre‑stimulation– explanation of study procedures, informed consent, application of physiological sensors (ErgoLAB), baseline STAI–S and 2‑minute rest.
ii. Randomized exposure to five sensory conditions — Control (sensors only, no plant stimuli), Visual, Tactile, Olfactory, and Tactile‑Olfactory — each lasting 4 min. The order was randomized across participants. Each exposure was followed by a 3‑minute STAI–S assessment and a 2‑minute rest (total ~ 9 min per condition). Participants wore a mask during all plant‑based conditions to control visual and nasal inputs. Physiological data (heart rate, HRV indices, skin conductance) were recorded continuously throughout.
iii. Post‑stimulation– sensors were removed and final STAI–S administered. Total session duration per participant was approximately 60 min. Participants were masked throughout all conditions (including Control), ensuring that mask-wearing did not vary and therefore did not influence the comparative results.

### Physiological measurements

Participants’ autonomic responses were continuously monitored using the ErgoLAB “Human–Machine–Environment” synchronization platform (Kingfar Inc., Beijing). This system comprises wireless, wearable sensors linked to a high-resolution data logger, with onboard preprocessing including low-pass, high-pass, and notch filtering to reduce noise artifacts during data capture [31].

#### Heart rate variability (HRV) metrics

HRV was recorded via a wireless photoplethysmogram (PPG) sensor attached to the participant’s earlobe, sampled at 64 Hz in accordance with device specifications [Kingfar ErgoLAB manual]. We analyzed average heart rate (HR) and both time-domain and frequency-domain HRV metrics to comprehensively assess autonomic nervous system dynamics. In the time domain, we calculated the standard deviation of NN intervals (SDNN) to assess overall variability in heartbeat intervals and the root mean square of successive differences (RMSSD), a robust measure of parasympathetic activity linked to relaxation and stress recovery [35]. For frequency-domain analysis, signals were filtered and log-transformed (natural log) to normalize the data. High-frequency (HF; 0.15–0.40 Hz) power reflects cardiac vagal tone (parasympathetic activity), whereas low-frequency (LF; 0.04–0.15 Hz) power reflects a mix of sympathetic and parasympathetic influences [35]. We computed the LF/HF ratio to index the balance of autonomic activity (higher LF/HF suggests relatively greater sympathetic influence [38, 39].

#### Skin conductance (SC)

SC, a measure of electrodermal activity (EDA) and sympathetic arousal, was recorded using the ErgoLAB platform with a wireless EDA sensor (electrodes on two fingers of the left hand). The sensor had a range of 0–30 µS, accuracy of 0.1 µS, and a sampling frequency of 32 Hz. Both Skin Conductance Level (SCL; tonic component) and Skin Conductance Responses (SCR; phasic component) were extracted. Signal processing included filtering and deconvolution techniques to minimize noise [40]. We extracted and analyzed Skin Conductance Level (SCL) — the tonic, baseline measure of autonomic arousal — using time-domain metrics. EDA reflects processes such as sweat gland activity and blood vessel dilation, offering real-time insights into arousal [41].

### Psychological measurements

#### State-trait anxiety inventory (STAI–S)

The 20-item State Anxiety subscale of the State–Trait Anxiety Inventory (STAI–S) [42] was administered immediately after each sensory condition to assess participants’ momentary anxiety [25, 43]. Items included statements such as ‘I feel tense,’ ‘I feel calm,’ and ‘I feel secure,’ rated on a 4-point scale from ‘not at all’ (1) to ‘very much so’ (4). Total scores range from 20 (lowest anxiety) to 80 (highest anxiety), with higher values indicating greater anxiety. The Trait subscale was not administered, as the focus was on acute emotional responses.

### Statistical analysis

Data were analyzed using IBM SPSS^®^ Statistics (Version 27). To improve normality, HRV parameters were natural log-transformed prior to analysis. A one-way repeated-measures ANOVA was then conducted to examine differences across the five sensory conditions. Normality was verified using Shapiro-Wilk tests on model residuals, and sphericity was tested via Mauchly’s test. Post hoc comparisons were adjusted using the Bonferroni correction to control for Type I error. Pearson’s correlation coefficients were calculated to explore associations between physiological and psychological variables. Statistical significance was set at 𝑝 < 0.05. Results are presented as mean ± standard deviation (SD), and visualizations were created using Origin 2022.

## Results

### Heart rate variability across sensory conditions

#### HR

**Fig. 3:**
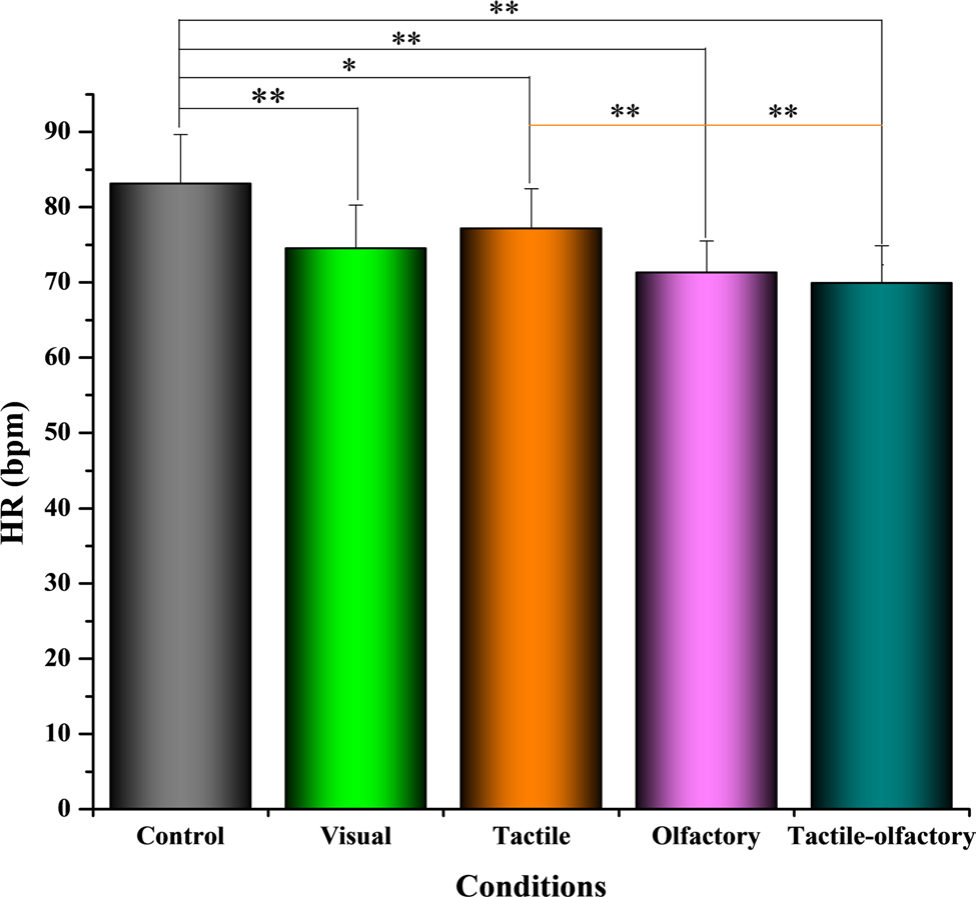
HR under five sensory stimulation conditions (bpm, beats per minute). Asterisks indicate significant differences based on Bonferroni-adjusted pairwise comparisons: *p* < 0.05 (*), *p* < 0.01 (**)

A one-way repeated-measures ANOVA revealed a significant main effect of condition on HR, *F*(4, 156) = 22.74, *p* < 0.001, partial η² = 0.487. The control condition had the highest mean HR (83.12 ± 6.53 bpm), followed by the tactile condition (77.16 ± 5.28 bpm) and visual condition (74.52 ± 5.76 bpm). The olfactory condition (71.32 ± 4.19 bpm) and tactile-olfactory condition (69.88 ± 4.97 bpm) showed the lowest HR values (Fig. 3), demonstrating the effectiveness of multisensory engagement in reducing physiological stress.

Post hoc pairwise comparisons with Bonferroni correction revealed several significant differences in HR across sensory conditions. HR in the control condition was significantly higher than in the visual (ΔM = 8.60, *p* < 0.001), tactile (ΔM = 5.96, *p* = 0.037), olfactory (ΔM = 11.80, *p* < 0.001), and tactile-olfactory conditions (ΔM = 13.24, *p* < 0.001). The tactile-olfactory condition showed significantly lower HR compared to control (ΔM = − 13.24, *p* < 0.001), tactile (ΔM = − 7.28, *p* < 0.001), and marginally lower HR than the visual condition (ΔM = − 4.64, *p* = 0.060), though this last difference did not reach statistical significance. No significant difference was found between the tactile-olfactory and olfactory conditions (ΔM = − 1.44, *p* = 1.000). HR in the olfactory condition was significantly lower than in control (ΔM = − 11.80, *p* < 0.001) and tactile (ΔM = − 5.84, *p* = 0.002), but not significantly different from the visual condition (ΔM = − 3.20, *p* = 0.385). Additionally, no significant difference was observed between the visual and tactile conditions (ΔM = − 2.64, *p* = 0.869). Among all conditions, the tactile-olfactory stimulus elicited the lowest HR, followed by olfactory and visual stimuli, while the control condition consistently produced the highest HR values. These findings highlight the superior physiological calming effects of combined tactile and olfactory stimulation relative to single-sensory or no-stimulus conditions.

#### LF/HF

**Fig. 4:**
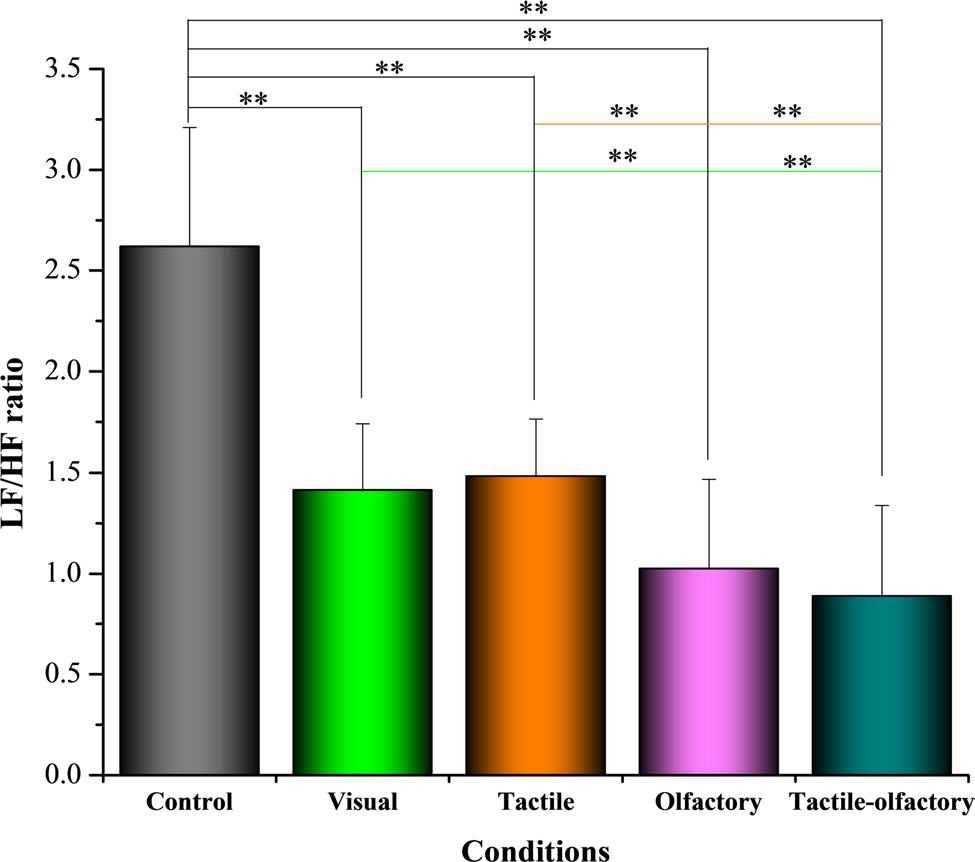
Mean LF/HF ratio under five sensory stimulation conditions. Asterisks indicate statistically significant differences based on Bonferroni-adjusted pairwise comparisons: *p* < 0.01 (**)

Statistical analysis revealed significant differences in the LF/HF ratio across the five sensory conditions *F*(4, 156) = 55.09, *p* < 0.001, partial η² = 0.697. The descriptive statistics showed that the control condition had the highest mean LF/HF ratio (2.62 ± 0.59), while the tactile-olfactory stimuli exhibited the lowest ratio (0.89 ± 0.45) (see Fig. 4), suggesting heightened vagal activity and greater relaxation in the latter condition.

Further analysis identified significant pairwise differences. The control condition showed significantly elevated LF/HF ratios compared to all other conditions (*p* < 0.001), including visual (ΔM = 1.205), tactile (ΔM = 1.138), olfactory (ΔM = 1.595), and tactile-olfactory (ΔM = 1.731). The tactile-olfactory condition demonstrated significantly lower LF/HF ratios compared to the visual (ΔM = − 0.526, *p* = 0.001), tactile (ΔM = − 0.593, *p* < 0.001), and olfactory conditions (ΔM = − 0.136, *p* = 1.000); however, the difference with olfactory was not statistically significant. Additionally, tactile stimulation yielded significantly higher LF/HF values than olfactory (ΔM = 0.457, *p* = 0.005). These results underscore the superior effectiveness of tactile-olfactory stimuli in promoting autonomic balance through enhanced parasympathetic activation and reduced sympathetic dominance.

#### SDNN

**Fig. 5:**
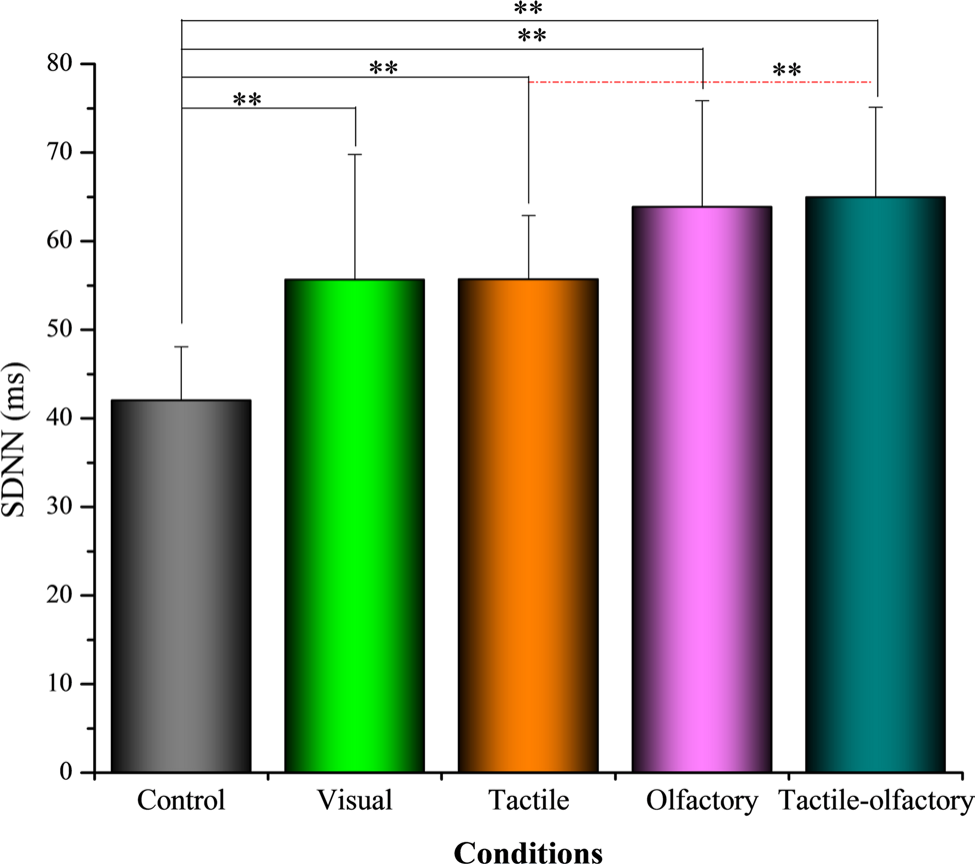
Mean SDNN (ms) under five sensory stimulation conditions. Asterisks indicate statistically significant differences based on Bonferroni-adjusted pairwise comparisons: *p* < 0.01 (**)

Figure 5 illustrates the distribution of SDNN values for each condition. A one-way ANOVA revealed significant differences in SDNN values across the five sensory conditions *F*(4, 156) = 20.12, *p* < 0.001, partial η² = 0.456. The descriptive statistics indicated that the control condition yielded the lowest average SDNN (41.99 ± 6.06), while the tactile-olfactory stimuli demonstrated the highest average SDNN (64.96 ± 10.17). This result suggests that the combination of tactile-olfactory stimuli is most effective in promoting physiological relaxation, as reflected by increased SDNN values.

Bonferroni-adjusted comparisons revealed that the control condition had significantly lower SDNN values than all other conditions: visual (ΔM = − 13.65, *p* = 0.002), tactile (ΔM = − 13.66, *p* < 0.001), olfactory (ΔM = − 21.83, *p* < 0.001), and tactile-olfactory (ΔM = − 22.96, *p* < 0.001). Tactile-olfactory stimuli showed significantly higher SDNN values than tactile (ΔM = 9.30, *p* = 0.012), but did not significantly differ from visual (ΔM = 9.31, *p* = 0.121) or olfactory (ΔM = 1.13, *p* = 1.000). The olfactory condition demonstrated significantly higher SDNN than control (ΔM = 21.83, *p* < 0.001), but did not significantly differ from visual (ΔM = 8.18, *p* = 0.372) or tactile (ΔM = 8.16, *p* = 0.118). These findings highlight the potential of multisensory stimulation, particularly tactile-olfactory engagement, in enhancing physiological relaxation as reflected by elevated SDNN.

#### RMSSD

**Fig. 6:**
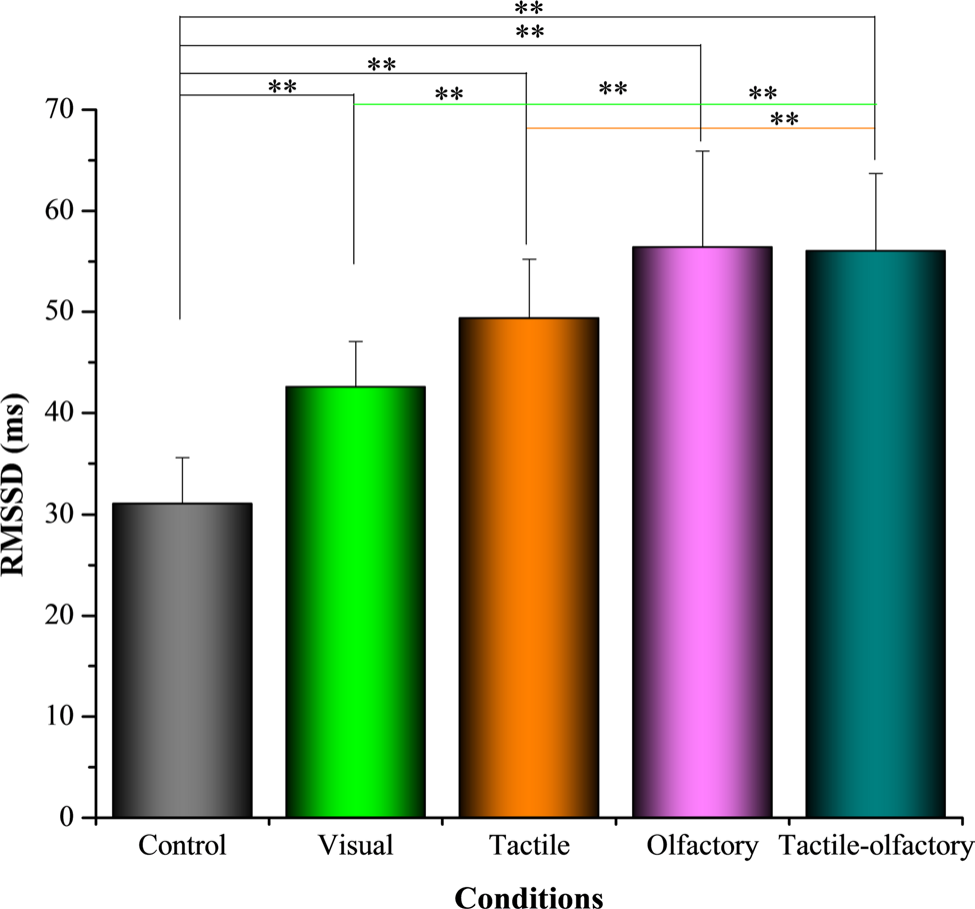
Mean RMSSD (ms) under five sensory stimulation conditions. Asterisks indicate statistically significant differences based on Bonferroni-adjusted pairwise comparisons: *p* < 0.01 (**)

ANOVA results showed significant variation in RMSSD values across the five sensory conditions *F* (4, 156) = 59.58, *p* < 0.001, partial η² = 0.713, indicating that different stimuli conditions influenced participants’ parasympathetic activity as measured by RMSSD. The descriptive analysis shows that the Control condition resulted in the lowest RMSSD values (31.05 ± 4.53), while the olfactory condition had the highest mean value (56.39 ± 9.54) (see Fig. 6). This highlights the significant restorative effect of the olfactory condition on physiological relaxation compared to the other conditions.

Bonferroni-adjusted post hoc tests indicated significant differences between the control condition and all other sensory conditions, confirming its significantly lower RMSSD values. Specifically, RMSSD in the control condition was significantly lower than in the visual (ΔM = − 11.50, *p* < 0.001), tactile (ΔM = − 18.32, *p* < 0.001), olfactory (ΔM = − 25.34, *p* < 0.001), and tactile-olfactory conditions (ΔM = − 24.97, *p* < 0.001). The olfactory condition also showed significantly higher RMSSD values than the visual condition (ΔM = 13.84, *p* < 0.001). However, no statistically significant differences were observed between the olfactory and tactile conditions (ΔM = 7.03, *p* = 0.10). These results confirm that multisensory engagement, particularly the olfactory and tactile-olfactory conditions, significantly enhance autonomic flexibility and promote relaxation.

### Skin conductance (SC)

**Fig. 7:**
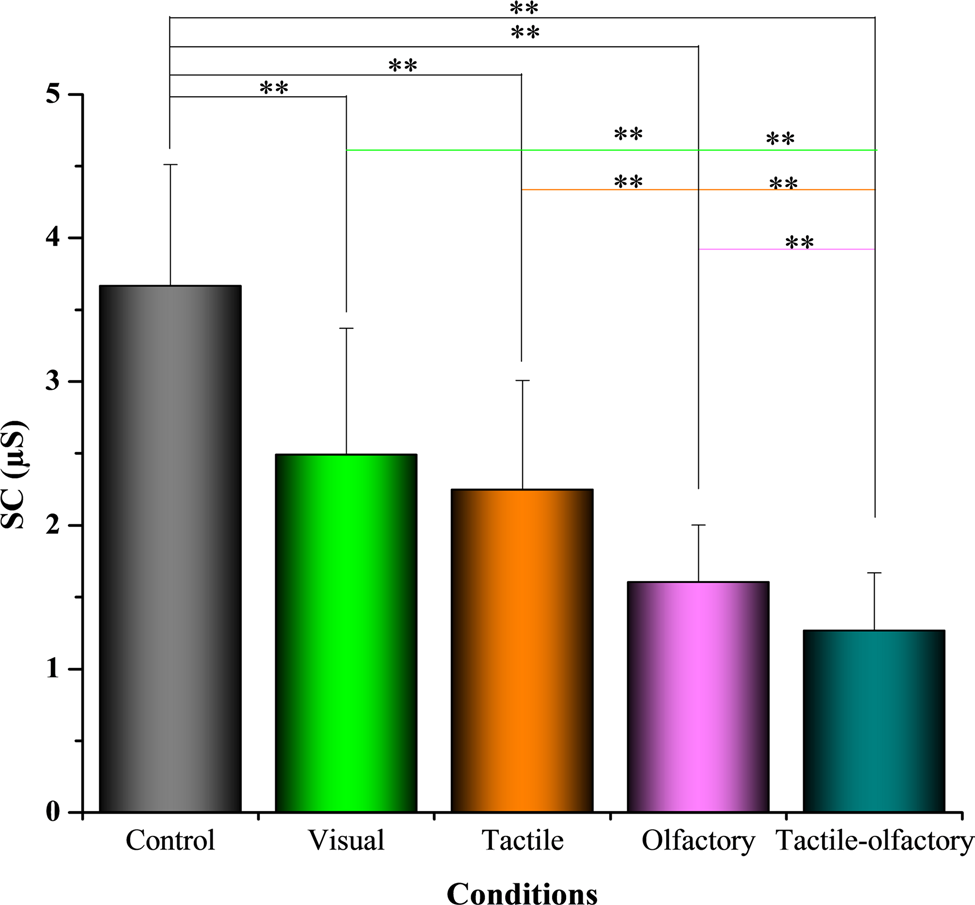
Mean skin conductance under five sensory stimulation conditions. Asterisks indicate statistically significant differences based on Bonferroni-adjusted pairwise comparisons: *p* < 0.01 (**)

Statistical analysis revealed significant differences in SC across the five sensory stimuli conditions *F*(4, 156) = 49.95, *p* < 0.001, partial η² = 0.675. Descriptive statistics indicated that the control condition had the highest average SC (3.67 ± 0.85), while the tactile-olfactory stimuli showed the lowest SC values (1.27 ± 0.40) (see Fig. 7), suggesting the greatest reduction in physiological arousal under multisensory stimulation. Pairwise comparisons showed that the control condition significantly differed from all other conditions: visual (ΔM = 1.18, *p* < 0.001), tactile (ΔM = 1.42, *p* < 0.001), olfactory (ΔM = 2.06, *p* < 0.001), and tactile-olfactory (ΔM = 2.40, *p* < 0.001). Among the experimental conditions, the tactile-olfactory condition demonstrated significantly lower SC compared to visual (ΔM = − 1.22, *p* < 0.001), tactile (ΔM = − 0.98, *p* < 0.001), and olfactory (ΔM = − 0.34, *p* = 0.017). These findings underscore that the tactile-olfactory condition was the most effective in reducing sympathetic arousal, as reflected by significantly lower SC values.

### Anxiety reduction measured by STAI–S scores

**Fig. 8:**
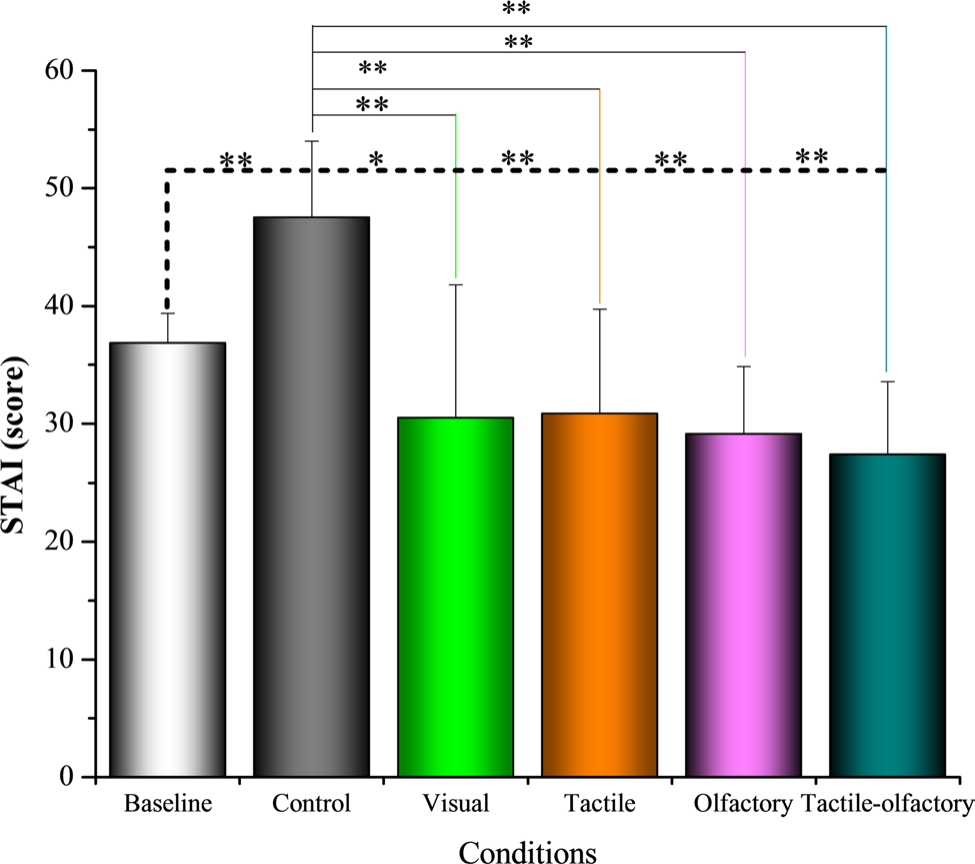
Mean STAI–S scores under five sensory stimulation conditions. Asterisks indicate statistically significant differences based on Bonferroni-adjusted pairwise comparisons: *p* < 0.05 (*), *p* < 0.01 (**)

Baseline state anxiety levels (STAI-S), assessed prior to any exposure, showed no significant differences across participants assigned to sensory conditions. Notably, anxiety levels significantly increased from baseline to the control condition (36.84 ± 2.54) to the control condition (47.52 ± 6.49), *p* < 0.001, confirming the stress-inducing nature of the control setting. A repeated-measures ANOVA revealed a significant effect of sensory condition on post-exposure anxiety levels, *F*(4, 156) = 25.32, *p* < 0.001, partial η² = 0.513. Descriptive statistics indicated that the control condition elicited the highest anxiety, significantly exceeding all other conditions. In contrast, the tactile-olfactory condition yielded the lowest anxiety (27.40 ± 6.16), suggesting multisensory integration substantially reduced state anxiety (Fig. 8).

Bonferroni-adjusted post hoc comparisons confirmed that the control condition differed significantly from all other experimental conditions, showing higher anxiety scores than the visual (ΔM = 17.04, *p* < 0.001), tactile (ΔM = 16.68, *p* < 0.001), olfactory (ΔM = 18.40, *p* < 0.001), and tactile-olfactory conditions (ΔM = 20.12, *p* < 0.001). However, no statistically significant differences were found among the experimental conditions themselves. For example, although tactile-olfactory elicited lower anxiety scores than visual (ΔM = 3.08), this difference was not statistically significant (*p* = 1.000). Likewise, comparisons between olfactory and tactile conditions yielded non-significant results. These results indicate that each sensory condition significantly reduced anxiety relative to Control. The tactile–olfactory condition produced the largest mean decrease, although differences among experimental conditions were not statistically significant.

### Correlations between physiological and psychological measures

The correlation analysis revealed significant relationships between physiological and psychological measures, offering insights into the interplay between autonomic nervous system activity, anxiety, and physiological arousal (Table 2). HR showed a strong positive correlation with the LF/HF ratio (*r* = 0.532, *p* < 0.001), suggesting that increased heart rate is associated with heightened sympathetic dominance. In contrast, Mean HR exhibited negative correlations with both SDNN (*r* = -0.505, *p* < 0.001) and RMSSD (*r* = -0.532, *p* < 0.001), indicating reduced heart rate variability (HRV) with elevated heart rates, reflecting decreased parasympathetic activity and greater stress.

**Table 2:** Correlation between physiological and psychological responses during and after the 4-min stimulation

	LF/HF	SDNN	RMSSD	SC	STAI–S
HR	0.532^**^	− 0.505^**^	− 0.532^**^	0.540^**^	0.433^**^
LF/HF		− 0.464^**^	− 0.671^**^	0.668^**^	0.549^**^
SDNN			0.527^**^	− 0.426^**^	− 0.465^**^
RMSSD				− 0.615^**^	− 0.483^**^
SC					0.525^**^

**. Correlation is significant at the 0.01 level (2-tailed)

The LF/HF ratio correlated positively with SC (*r* = 0.668, *p* < 0.001) and STAI–S (*r* = 0.549, *p* < 0.001), suggesting that sympathetic dominance is aligned with increased physiological arousal and higher subjective anxiety. Conversely, LF/HF was negatively associated with SDNN (*r* = -0.464, *p* < 0.001) and RMSSD (*r* = -0.671, *p* < 0.001), further supporting the role of reduced HRV in stress responses.

HRV parameters, SDNN and RMSSD, displayed strong positive intercorrelations (*r* = 0.527, *p* < 0.001), with both negatively correlating with SC (SDNN: *r* = -0.426, *p* < 0.001; RMSSD: *r* = -0.615, *p* < 0.001) and STAI–S (SDNN: *r* = -0.465, *p* < 0.001; RMSSD: *r* = -0.483, *p* < 0.001). These findings highlight the link between increased HRV and reduced anxiety or physiological arousal. SC positively correlated with STAI–S (*r* = 0.525, *p* < 0.001), further indicating the association between heightened physiological arousal and anxiety. These results indicate that under the tactile-olfactory stimuli, physiological and psychological states are closely interconnected.

## Discussion

Creating calming, anxiety-reducing environments in healthcare settings, particularly in hospitals and outpatient clinics, remains a critical challenge due to their association with heightened stress and discomfort. This study addresses a critical gap in healthcare research by demonstrating the transformative potential of multisensory engagement—tactile, olfactory, and visual stimuli—as a natural, non-invasive intervention to mitigate anxiety in clinical environments. By utilizing these sensory modalities in a dental clinic setting, this research provides novel insights into how human interaction with natural elements can regulate autonomic responses and foster physio-psychological well-being, particularly in high-stress environments.

Our results showed that engaging both touch and smell with plants significantly reduced anxiety and stress markers in a dental-clinic context. The combination of tactile-olfactory stimuli demonstrated superior calming effects, as evidenced by reduced anxiety scores and enhanced parasympathetic activity compared to other conditions. This supports the role of sensory integration in modulating emotional and physiological responses, as emphasized in previous studies [26, 44]. Moreover, it aligns with SRT, and ART, which suggest that humans have an innate preference for natural environments, as shaped by evolution, and that exposure to nature activates the parasympathetic nervous system, promoting both psychological and physiological recovery from stress [45, 46]. While previous research has primarily examined single-modality interventions, such as visual or auditory stimuli [47], with limited attention to tactile and olfactory inputs. This study addresses this gap by investigating the combined effects of tactile and olfactory stimuli, which remain underexplored in stress-reduction research. Furthermore, the results emphasize the unique contributions of touch and scent, which remain underexplored in a body of literature dominated by visual-centric approaches [48, 49]. These findings suggest that healthcare environments could be enhanced by incorporating underutilized sensory modalities beyond vision. In particular, tactile and olfactory inputs showed notable benefits.

### Cardiovascular health indicators

The physiological benefits of multisensory engagement were most evident in HRV metrics, which are reliable indicator of autonomic function [50]. Our findings showed that tactile-olfactory and olfactory conditions significantly reduced heart rate (HR) and LF/HF ratio compared to the control, reflecting enhanced PNS activation and reduced sympathetic dominance [51]. In contrast, the control condition exhibited elevated HR and LF/HF ratios, indicative of heightened physiological stress. While visual and tactile conditions alone produced less pronounced effects, they still outperformed the control, suggesting that even isolated exposure to greenery through non-visual modalities can contribute to autonomic regulation. These findings reinforce the importance of incorporating sensory interventions in clinical settings to restore autonomic balance and reduce stress-related physiological responses.

Beyond HR and LF/HF ratios, HRV parameters such as SDNN and RMSSD increased significantly under the tactile–olfactory and olfactory conditions, indicating improved physiological resilience to stress [52]. Higher SDNN and RMSSD values are typically associated with increased parasympathetic activity, reflecting states of recovery and relaxation [53]. These results are consistent with previous literature demonstrating the therapeutic potential of natural elements in enhancing cardiac vagal activity and supporting autonomic balance [20, 22]. The reductions in LF/HF ratio observed under sensory-rich conditions are consistent with increased parasympathetic (relaxation) activity, as reported in prior literature [47, 54]. These cardiovascular responses build upon earlier studies linking nature exposure to physiological relaxation. For example, Franco et al. (2017) reported reductions in HR and SC in response to natural environments [26], while Elsadek et al. (2019) highlighted the role of greenery in modulating autonomic activity [20]. Unlike previous studies focused on visual stimuli alone, the present findings emphasize the additive and potentially synergistic effects of combining tactile and olfactory sensory inputs. This integrated approach illustrates how combining sensory stimuli may produce stronger physiological relaxation than visual stimuli alone.

These results support the benefits of plant-based multisensory stimuli for autonomic regulation under stress. The observed increase in parasympathetic nervous system (PNS) activity—associated with reduced heart rate, blood pressure, and physiological arousal [55], is consistent with prior research [8]. Concurrently, the decrease in sympathetic nervous system (SNS) activity aligns with findings that link elevated SNS indices to heightened stress [56–58]. The notable reduction in LF/HF ratios under sensory-rich conditions reinforces the therapeutic role of nature-based interventions in restoring autonomic balance and promoting well-being in clinical settings [48].

In addition to HRV, SC measurements offered complementary evidence of sympathetic arousal modulation. Elevated SC values observed in the control condition reflected heightened arousal, whereas significant reductions in SC were observed under the olfactory and tactile–olfactory conditions [59]. Interestingly, the olfactory condition alone produced reductions comparable to the combined condition, highlighting the potency of scent-based interventions. This finding is supported by research on the neurophysiological mechanisms of olfactory stimuli, particularly their activation of the limbic system and regulation of mood and autonomic responses through regions such as the hypothalamus and hippocampus [60–63]. The therapeutic role of fragrant plants in clinical settings—rooted in the historical concept of healing gardens—was reaffirmed in our study [64]. Thus, incorporating aromatic plants into healthcare environments represents a scalable and culturally adaptable strategy for reducing sympathetic arousal and fostering autonomic recovery.

### Psychological benefits of sensory interventions

The significant reduction in STAI–S scores, particularly under tactile-olfactory conditions, demonstrates the psychological benefits of multisensory engagement observed in this study. According to normative STAI–S benchmarks, scores of 20–37 represent low anxiety, 38–44 moderate, and 45–80 high anxiety [65]. The control condition in our study averaged 47.5, placing participants in the high anxiety range, consistent with the anticipatory stress often experienced in dental waiting environments. The tactile-olfactory condition, by contrast, reduced average scores to 27.4—within the low anxiety range—reflecting a ~ 42% reduction. These results underscore the clinically meaningful impact of sensory stimulation.

Consistent with prior research [19, 66, 67], the inclusion of indoor plants and sensory-rich stimuli significantly reduced anxiety levels, with the tactile-olfactory and olfactory conditions proving particularly effective. These findings align with broader evidence on the role of sensory interventions in alleviating anxiety and fostering emotional well-being, underscoring their potential for addressing procedural stress in healthcare [26]. Similarly, prior studies have shown that incorporating indoor plants can reduce negative emotions and foster psychological comfort [68, 69], further supporting the therapeutic potential of nature-based, multisensory interventions in high-stress healthcare settings. Notably, while visual stimuli provided measurable benefits, they elicited smaller parasympathetic responses compared to tactile or olfactory engagement. This contrast highlights the unique effectiveness of tactile and olfactory inputs, emphasizing the need to leverage these underutilized modalities in designing stress-reducing healthcare environments.

### Correlations between physiological and psychological measures

The observed correlations between HRV metrics, SC, and STAI–S scores provide strong evidence for the interplay between autonomic nervous system activity and subjective anxiety levels. For example, reduced HRV and elevated SC values were strongly correlated with heightened anxiety, whereas conditions enhanced parasympathetic activity were linked to improved psychological outcomes [8]. These correlations reinforce the utility of HRV and SC as biomarkers for evaluating the effectiveness of sensory interventions in clinical settings.

Recent advancements in neurophysiological research have provided valuable insights into the mechanisms underlying stress regulation. Studies indicate that the prefrontal cortex plays a central role in modulating autonomic function, particularly parasympathetic activity, which is closely associated with relaxation and emotional stability [70]. Neuroimaging studies, such as those using Electroencephalography and functional magnetic resonance imaging, have revealed that urban environments elicit stronger amygdala activation compared to natural settings, highlighting the impact of environmental stimuli on mood and ANS balance [17, 71]. These findings support the notion that sensory-rich natural environments may engage brain regions critical for anxiety and stress regulation, leading to adaptive changes in ANS responses.

Tailoring multisensory interventions to enhance parasympathetic activity and minimize sympathetic dominance offers a promising framework for creating stress-reducing environments. This approach addresses procedural anxiety while contributing to broader efforts aimed at improving patient comfort and the overall healthcare experience.

### Practical implications for healthcare design

The demonstrated benefits of tactile and olfactory stimuli suggest their integration into healthcare design, such as using fragrant plants and touchable greenery in waiting areas, to create calming atmospheres that reduce procedural anxiety and improve patient satisfaction. Nature-based multisensory interventions offer low-cost, culturally adaptable solutions for improving patient comfort and reducing stress in diverse healthcare settings. Although close-range plant interactions were employed to ensure reliable multisensory engagement under controlled conditions, this configuration should be viewed as a scientific exploration rather than a prescriptive design model. For practical application, we propose design adaptations such as embedding low-maintenance aromatic plants in accessible planter installations, diffusing plant-based scents in waiting areas, and incorporating biotextural elements (e.g., foliage, bark) into architectural surfaces.

### Study limitations and future directions

While the study provides valuable insights, several limitations should be acknowledged. First, the participant pool primarily consisted of young adults, whose heightened autonomic adaptability may have amplified the observed effects. Future studies should include older adults or individuals with clinical anxiety to assess whether similar physiological and psychological responses emerge. Second, although the experiment was conducted in a real dental clinic, participants were not undergoing invasive procedures, which may limit ecological realism. Third, the study excluded auditory and gustatory modalities to streamline the design. Future research should explore the integrative effects of these additional senses to build a more comprehensive understanding of multisensory stress regulation. Fourth, cultural differences in olfactory and tactile preferences may influence affective and autonomic responses; thus, cross-cultural replication is warranted. Finally, this study focused on short-term responses. Longitudinal research is needed to examine the durability and cumulative benefits of repeated exposure in diverse clinical contexts.

## Conclusion

This study demonstrates that multisensory engagement—particularly the combination of tactile and olfactory stimuli—can significantly reduce anxiety and enhance physiological relaxation in a dental clinic setting. Compared to the control, participants exposed to plant-based sensory stimuli showed marked improvements in heart rate, heart rate variability (LF/HF, SDNN, RMSSD), skin conductance, and STAI–S scores. These findings provide preliminary evidence for integrating targeted, nature-inspired sensory stimuli (especially scent and touch) into healthcare waiting areas to improve patient comfort and reduce anxiety. By grounding design strategies in physiological and psychological evidence, this study contributes to improving patient experience in high-stress settings.

## Data Availability

Data is provided within the manuscript.
